# Mental Illnesses and Stigma among Medical Undergraduates in India

**DOI:** 10.5334/aogh.4523

**Published:** 2024-10-29

**Authors:** Semanti Das, Sunom Merab Lepcha, Ashish Pundhir, Senthil Amudhan

**Affiliations:** 1 Senior Resident, Centre for Community Medicine, All India Institute of Medical Sciences, New Delhi, India; 2 Senior Resident, Department of Community Medicine and Family Medicine, All India Institute of Medical Sciences Kalyani, Naidia, West Bengal, India; 3 Assistant Professor, Department of Community Medicine and Family Medicine, All India Institute of Medical Sciences Kalyani, Naidia, West Bengal, India; 4 Professor and Head, Department of Epidemiology, National Institute of Mental Health and Neuro Sciences, Bangalore, India

**Keywords:** Mental health, Medical Students, stigma

## Abstract

The current Medical Education system of India, with its enormous workload and academic demands tend to cause the medical students stress. There is evidence showing medical students at a higher risk of depression, anxiety and burnout compared to other Indian students. Despite, the huge reported numbers, the proportion of students and doctors who seek help for their problems is alarmingly low. One of the reasons provided for the same is stigma towards mental health and an apprehension regarding labels and treatment history on the careers of the students.

Increased awareness and with a National Health Programme catered towards mental health, there has been a boost in the utilization and provision of mental health services but it rarely translates into better mental health facilities for the healthcare providers.

The special set of challenges faced by a medical students are gradually being recognized and efforts are being made to address them. Curriculum guidelines, teaching methods, student welfare centres and helplines have been the areas of intervention. There should also be changes in approaches towards the students who face problems and providing a safe environment for them to discuss their problems, including encouraging peer support.

Thus, a fine balance needs to be present between ensuring the protection of the mental health of a medical student and ensuring a quality medical education for them. Further exploration to address stigma and building empathy among the students and evaluation of the intervention methods devised to address the same becomes very necessary to ensure fruitful interventions.

It is the need of the hour to help Indian Medical students overcome their struggles with mental health.

The Indian medical education curriculum involves a time‑ and discipline‑based approach designed to train undergraduates to become competent and skilled clinicians. The current education structure is spread over five years and aims to develop a student’s basic knowledge and skillset. The vast and ever‑developing medical science requires a huge deal of motivation and continual unwavering efforts on the part of the medical students. The enormous workload involved, the academic demands, preoccupation with clinical experience, worry about the future, and the challenging learning experiences that students come across during medical training often result in psychological distress among students [1].

A meta‑analysis‑based study estimated the prevalence of depression among medical students in India to be 50% (confidence interval (CI): 32%–47%) [2], the prevalence of anxiety disorder to be 34.5% (10.1–58.9%) [3], and the risk for suicidal behavior to be 29.6–53.6% [4, 5] with 2.6% having attempted suicide at least once in their life [5]. Burnout was also found to be a common occurrence, with the prevalence reported being 16.84–80% [6, 7].

Despite this huge burden reported, the rate of health‑seeking behavior for mental illnesses in medical students is alarmingly low [8]. Untreated mental illnesses in medical students and, in turn, doctors can be potentially disastrous, impacting the quality of life not only of the students themselves but also the quality of care of the patients they would treat in due course of time. Stigma is one of the main barriers to treatment seeking by medical students [4]. Fear of impact on career, judgment, and discrimination have been found to affect health‑seeking behavior among medical students.

With improved awareness regarding mental health, India has geared toward reducing the stigma around mental health through a national‑level program. It acts to protect and safeguard the rights of people with mental illnesses. Helplines and telemedicine interventions have also paved the pathway for greater accessibility of health services. However, the efforts have not translated well into ensuring the providers’ mental health.

Improving awareness of mental illnesses and their impact on medical students needs momentum to normalize seeking help for mental health. The medical education environment in India requires a tailored approach to handling the specific problems faced by Indian medical students. The National Medical Council (NMC) is the central regulatory body for medical education and professionals. Regulation of duty hours and suicide prevention measures focused on suicidal act deferral have been essential action points. Student wellness centers currently serve as the point of care and a safe space for the mental health struggles of medical students. The council has also envisaged guidelines for objective conduct of medical professional examinations to reduce bias and unfair marking. The NMC has also convened a committee to investigate the high prevalence of potentially risky mental health ailments among medical students. The current scenario requires cognizance of the problem through a broader perspective with national‑level estimates. National‑level surveys and free and empowered talks among medical students regarding mental health are the need of the hour.

Implementing the measures to help medical students is beset with challenges. The effects of competition and the academic excellence expected from medical students can be offset only with a focused and spaced curriculum focused on understanding and moving away from information retention. Many institutions do not follow duty hour regulations due to the patient load. There is a lack of awareness about the signs and symptoms of burnout and an empathetic approach after the diagnosis. Helpline numbers are the deferral points and do not help in preventing further attempts. Often, a student does not have the time or energy left to acknowledge their mental health condition before it is too late. Another potential obstacle for medical students is that the mental health issues of the student are attributed to the academic conditions and the departmental environment, thus potentially antagonizing the faculty if the student comes forward with their problems.

It is imperative that medical students are provided with an environment conducive to their mental health. Strengthening student welfare services is essential to reduce stigma and promote help‑seeking behaviors. A peer support approach is also crucial in reducing stigma. Public or professional stigma reduction interventions must also measure the impact of such interventions using fit‑for‑purpose, psychometrically sound instruments to provide a holistic benefit to the students. Finally, medical students, with their orientation toward building empathy for their patients, require a more empathetic and supportive environment for discussing and overcoming struggles with their mental health.

## References

[r1] Grover S. Mental health issues among medical students. J Ment Health Hum Be. 2022;27(2):71. doi:10.4103/jmhhb.jmhhb_297_22

[r2] Dwivedi N, Sachdeva S, Taneja N. Depression among medical students of India: Meta‑analysis of published research studies using screening instruments. Indian J Soc Psychiatry. 2021;37(2):183. doi:10.4103/ijsp.ijsp_119_20

[r3] Sarkar S, Gupta R, Menon V. A systematic review of depression, anxiety, and stress among medical students in India. J Ment Health Hum Be. 2017;22(2):88. doi:10.4103/jmhhb.jmhhb_20_17

[r4] Arun P, Ramamurthy P, Thilakan P. Indian medical students with depression, anxiety, and suicidal behavior: Why do they not seek treatment? Indian J Psychol Med. 2022;44(1):10–16. doi:10.1177/0253717620982326 35509655 PMC9022918

[r5] Goyal A, Kishore J, Anand T, Rathi A. Suicidal ideation among medical students of Delhi. J Ment Health Hum Be. 2012;17:60–69.

[r6] Pharasi S, Patra S. Burnout in medical students of a tertiary care Indian medical center: How much protection does resilience confer? Indian J Psychiatry. 2020;62(4):407–412. doi:10.4103/psychiatry.IndianJPsychiatry_681_19 33165365 PMC7597710

[r7] Farrell SM, Kar A, Valsraj K, et al. Wellbeing and burnout in medical students in India; a large scale survey. Int Rev Psychiatry. 2019;31(7–8):555–562. doi:10.1080/09540261.2019.1688047 31774379

[r8] Seera G, Arya S, Sethi S, Nimmawitt N, Ratta‑apha W. Help‑seeking behaviors for mental health problems in medical students: Studies in Thailand and India. Asian J Psychiatr. 2020;54:102453. doi:10.1016/j.ajp.2020.102453 33271732

